# A Neglected Part of Education

**Published:** 1881-07

**Authors:** J. J. Keyes


### A NEGLECTED PART OF EDUCATION.

BY J. J. KEYES.

What *is* education? The proper, original idea of the word educate is, to "lead out." Applied to man, it would mean to lead out, discipline and develop his various attributes of mind and body. And here I wish to remark that, in my opinion, any system of education that leaves entirely out of view the student's body is defective, and a great injustice to the student's mind. Whatever may be the constitution of man in a coming life, we very well know what it is in *this* life; and to treat him fairly, and to properly adapt the methods of his education to his condition and necessities, we must have regard to his whole nature. And if I speak almost exclusively of his physical nature, and of the method that ought to be pursued in relation to to it, it is because I think that part of his nature has been too much neglected. His nature, as we all know, is three fold, moral, mental and physical, or, spiritual, intellectual and animal. And yet, his training, hitherto, has been chiefly intellectual, a little moral, and hardly at all physical; and this, in my view, is a practical injustice and wrong; especially if you wish to educate men broadly, to a lofty virtue, and to such a development as will secure a proper balance and harmony of all their powers, and thus, their truest welfare. Persons in quest of an education should learn, not only what are their mental powers, and how properly to use them; what are their moral powers, and how properly to use them, but what are their physical powers, or animal conditions, and how properly to use them. I feel quite safe in affirming that man's greatest trouble with himself, the troubles he has with his fellow man, a large proportion of the vices and crimes that afflict society, and all the vast social ferment and disturbance that come from the relations of the sexes, arise, very largely, from defective systems of education, from a shameful neglect of physical culture. We have thought, seemingly, to cultivate the highest nature, and let the lower nature take care of itself; and in taking care of itself it has, as might have been expected, abused itself, and brought to society this great mischief and harm. But I will not be unfair. I know it is not that the animal, or lower nature, so called, has been entirely forgotten or overlooked. But it has been assumed by educators, theologians and preachers alike, that only by the culture of the intellectual and moral faculties, only by improving the mind and the conscience, and possibly the æsthetic tastes, could the animal nature be kept in proper trim and subjection. And right here, I think, is where they have made a tremendous mistake. The splendid physical condition of many of our western savages should have taught them better. They have educated men, and converted them, and brought them into the churches. The school-teachers and the ministers have each done their work; but what have they made? I admit, with thankfulness, that they have furnished us with many of the finest specimens of humanity. I admit that more frequently in the churches and in educated communities than anywhere else, you will find men and women of purity, temperance and self-control, who observe on principle, and from choice, the so-called proprieties of life. But does not any pastor, any educator, any citizen of much observation, having a general acquaintance with men, know that the lower nature of men, the animal, the physical, with its various appetites and passions, exerts a most deleterious, hurtful, influence over the lives and characters of a vast majority of professedly good men, religious men and members of churches? Yes, they do know it, and physicians know it, too, and could, if they would, make some very sad and startling revelations in almost any community. And for my part, instead of being astonished at the sins, and slips, and corruptions of society, in the churches and out of them, I wonder that they are not more numerous and more abominable than they are. Either a lack of knowledge, or a false delicacy seems to render impossible right instruction and proper training on the part of parents, teachers and preachers. I know it is customary for most people to attribute what I am thinking of entirely to the depravity of the human heart, to be cured only by religion. But this, to me, is a mere makeshift. And, as I have shown, religion, alone, does not seem to be a sufficient remedy, unless we can learn to understand the term broadly, as comprehending the threefold culture that our threefold nature requires. And, furthermore, to say that human depravity is accountable for the instincts, and appetites, and propensities of our animal nature, is to affirm that man is not now constituted, physically, as he was before the fall. And this it would be very difficult to show. Some of my readers are trying to get an education. I would have you know what a good education comprehends, and what price you must pay for it. First, you have an intellect—a mind. Of course any branch of study faithfully pursued will have a tendency to enlighten and develop your mental faculties. But there are studies specially adapted to this end. These are what engage your attention in the schools: mathematics, astronomy, natural philosophy, intellectual philosophy, logic, rhetoric, history. They educate you; that is they lead *out*, discipline, enlarge, enlighten the mind. But books are not enough. No man was ever properly and truly educated by the study of books alone. Many a man of very limited school advantages, but of shrewd observation, inquiring mind, retentive memory, reflecting judgment and common sense—which, by the way, is so uncommon, has become more truly educated than many another man who has had the rarest opportunities. You must study men. You must study nature—face to face. You must study governments, institutions and political economy. And besides, you must have a *long* and perhaps *severe* tuition under old mother experience—one of the best school mistresses the children of men ever had. But we have a *moral* nature too. This must not, *should* not be neglected. We should study moral science. We should cultivate our moral senses. We should listen to conscience. We should accustom ourselves to the study of moral questions. We should make a study of natural right and justice.

And then, lastly, we have a physical nature, called the animal, and sometimes the lower nature. It needs educating, or, rather, it needs tempering, moderating, controlling, developing, purifying; and we need educating with special reference to this needed process. Now what is the price of a good physical education, of good physical culture? Is it two hours practice in a gymnasium, in walking, in horseback riding, in sawing wood, or in house work, every day. (For I am writing for ladies as well as gentlemen.) These things, one or several, are good, and had better not be neglected. Is it the study of physiology, phrenology and biology? Is it the study of dietetics and hygiene? Yes, it is all these, and the study of chemistry, too, and many other things.

Since none of the constituent elements and forces of nature are wanting in the human body, see what intimate relations man sustains to nature. As to his body he is a child of nature, and must subsist wholly upon nature; and to do so, in health and comfort, he must live in harmony with nature. He must not expect to violate with impunity any law of nature, nor must he expect any special interposition of Providence to shield him from the consequences of disobedience. Of course, then, he should know something of physiology, biology, chemistry, dietetics, hygiene and anatomy, for these sciences tell him what he is made of, how he is put together, the relations of the various organs and members of the body to each other, and the functions of each, the laws of life and health, what he should eat and drink to build up, strengthen and keep in good repair the several parts of the human system. Think of the world of good that might come to mankind as the result of this knowledge. Nine-tenths of the disease, sickness and poor health that exist in the world to-day exist for the lack of it. Nine-tenths of all the physical weakness and disability found among men and women, boys and girls, exist for the same reason And a large part of the inebriety, also, that is the peculiar curse of our day. Think you, if our boys were faithfully and thoroughly instructed in our schools upon the real nature and merits of alcohol, and its certain fatal effects upon the human constitution, the method of its deadly work, and just what it is sure to do for blood, tissue, muscle, brain, heart, lungs, stomach, liver and nerves—think you, if thus intelligently posted and forewarned, so many of them would topple into dishonored graves as now do! I do not believe it. I believe God may look upon this generation and say, as in Hosea's time, "My people are destroyed for lack of knowledge."

